# Episodic Recollection Difficulties in ASD Result from Atypical Relational Encoding: Behavioral and Neural Evidence

**DOI:** 10.1002/aur.1448

**Published:** 2015-01-28

**Authors:** Sebastian B. Gaigg, Dermot M. Bowler, Christine Ecker, Beatriz Calvo‐Merino, Declan G. Murphy

**Affiliations:** ^1^ Department of PsychologyCity University LondonLondonEC1V0HBUK; ^2^ Institute of PsychiatryKing's College LondonLondonSE58AFUK

**Keywords:** autism, relational memory, item memory, recollection, familiarity

## Abstract

Memory functioning in Autism Spectrum Disorder (ASD) is characterized by impairments in the encoding of *relational* but not *item* information and difficulties in the *recollection* of contextually rich episodic memories but not in the retrieval of relatively context‐free memories through processes of *familiarity*. The neural underpinnings of this profile and the extent to which encoding difficulties contribute to retrieval difficulties in ASD remain unclear. Using a paradigm developed by Addis and McAndrews [2006; Neuroimage, 33, 1194–1206] we asked adults with and without a diagnosis of ASD to study word‐triplets during functional Magnetic Resonance Imaging (fMRI) scanning that varied in the number of category relations amongst component words. Performance at test confirmed attenuated recollection in the context of preserved familiarity based retrieval in ASD. The results also showed that recollection but not familiarity based retrieval increases as a function of category relations in word triads for both groups, indicating a close link between the encoding of relational information and recollection. This link was further supported by the imaging results, where blood oxygen level dependent (BOLD) signal responses in overlapping regions of the inferior prefrontal cortex were sensitive to the relational encoding manipulation as well as the contrast between recollection versus familiarity based retrieval. Interestingly, however, there was no evidence of prefrontal signal differentiation for this latter contrast in the ASD group for whom signal changes in a left hippocampal region were also marginally attenuated. Together, these observations suggest that attenuated levels of episodic recollection in ASD are, at least in part, attributable to anomalies in relational encoding processes. ***Autism Res*** *2015, 8: 317–327*. © 2015 International Society for Autism Research, Wiley Periodicals, Inc.

## Introduction

The broader cognitive profile of Autism Spectrum Disorder (ASD) is now well known to include a pattern of memory difficulties that holds clues to the neuropathology underlying the disorder and has important implications for the design of effective educational programs [see Boucher & Bowler, 2008; Bowler, Gaigg, & Lind, 2011; Boucher, Mayes, & Bigham, 2012; Gaigg & Bowler, 2012 for comprehensive reviews]. Briefly, working memory poses some difficulties for individuals with ASD when tasks probe the maintenance of progressively more numerous spatial locations [Morris, Rowe, Fox, Feigenbaum, Miotto, & Howlin, 1999; Steele, Minshew, Luna, & Sweeney, 2007; Williams, Goldstein, Carpenter, & Minshew, 2005] or when the unaided retrieval of the precise order of events is required [Poirier, Martin, Gaigg, & Bowler, 2011; Gaigg, Bowler, & Gardiner, 2013]. When demands go beyond the limited capacity of working memory, unaided free recall tends to be compromised, particularly for material that can be organized conceptually [Bowler, Matthews, & Gardiner, 1997; Bowler, Gaigg, & Gardiner, 2009, 2010; Cheung, Chan, Sze, Leung, & To, 2010; Gaigg, Gardiner, & Bowler, 2008; Tager‐Flusberg, 1991], or when learning is assessed over multiple trials [Bennetto, Pennington, & Rogers, 1996; Bowler, Gaigg, & Gardiner, 2008a; Minshew & Goldstein, 2001]. When individuals with ASD do recall previously encountered material they frequently fail to retrieve contextual details associated with the study episode such as where, when, how or from whom they have learned a particular fact [Bowler, Gardiner, & Berthollier, 2004; Hala, Rasmussen, & Henderson, 2005; Lind & Bowler, 2009; O'Shea, Fein, Cillessen, Klin, & Schultz, 2005; Russell & Jarrold, 1999]. Their recall of autobiographical memories also tends to be relatively void of contextual details that characterizes the personally experienced past [Crane & Goddard, 2008; Crane, Goddard, & Pring, 2009; Goddard, Howlin, Dritschel, & Patel, 2007; Lind & Bowler, 2010; Millward, Powell, Messer, & Jordan, 2000]. Contrasting these difficulties on tests of unaided recall, the performance of individuals with ASD on supported test procedures tends to be generally unaffected. Thus, tasks using rhymes [Tager‐Flusberg, 1991], word fragments [Boucher & Warrington, 1976; Bowler, Matthews, & Gardiner, 1997; Gardiner, Bowler, & Grice, 2003], category labels [Bowler, et al., 2009; Mottron, Morasse, & Belleville, 2001] or paired associates [Gardiner, et al., 2003; Minshew & Goldstein, 2001] as cues to previously studied material generally yield preserved levels of performance in ASD. Similarly, tests of recognition memory that require participants to discriminate studied from novel stimuli pose relatively few difficulties [Barth, Fein, & Waterhouse, 1995; Beversdorf, et al., 2000; Boucher, Cowell, Howard, Broks, Mayes, & Roberts, 2005; Bowler, Gaigg, & Gardiner, 2008b; Bowler, Gardiner, & Grice, 2000; Bowler, Gardiner, & Gaigg, 2007; Salmond, Ashburner, Connelly, Friston, Gadian, & Vargha‐Khadem, 2005].

The pattern of performance across supported and unsupported test procedures is indicative of relatively greater impairments in the retrieval than the encoding of information and has led Bowler et al. [2014, 2004] to formulate the “Task Support Hypothesis” according to which performance decrements in ASD can be alleviated by procedures that scaffold particularly memory retrieval. There are, however, important exceptions in the relevant literature. First, some studies report attenuated performance on cued recall and recognition tests by individuals with ASD [Bowler et al.^2014^, ^2004^; Chen et al.,^2014^, ^2009^; Scherf, Behrmann, Minshew, & Luna, 2008]. Second, when overall recognition performance is preserved, individuals with ASD consistently report fewer experiences of recollecting contextual information associated with the items they recognize, reporting a sense of familiarity that is contextually relatively void instead [Bowler et al.^2014^, ^2000^; Bowler et al.^2014^,^2014^, ^2007^]. Finally, when recognition is tested for specific combinations of items or item features, individuals with ASD perform significantly worse [Bowler, Gaigg, & Gardiner, 2014]. These exceptions indicate that certain encoding processes may also be compromised in ASD, which is further supported by studies that manipulate encoding conditions whilst holding retrieval conditions relatively constant [e.g., Gaigg et al., 2008; Mottron, et al., 2001; Toichi & Kamio, 2002]. In particular, the encoding of relations between items and between items and their contexts (*relational* information) appears to be compromised in ASD while the encoding of *item‐specific* information, including physical as well as conceptual features of items (e.g., that a banana is a *curved, yellow fruit*) is relatively preserved [Bowler, et al., 2011; Gaigg et al., 2008].

Disentangling encoding from retrieval processes is notoriously difficult because we inevitably retrieve information about the material we encode and we (re)encode material when we retrieve it. Nevertheless, elegant behavioral experimentation and advances in neuroimaging methods have led to a relatively detailed understanding of the functional organization of the human declarative memory system including the contributions of encoding and retrieval processes to performance on various memory tasks. Briefly, during encoding enthorhinal (ErC) and perirhinal (PrC) cortices of the medial temporal lobe (MTL) are thought to process information specific to individual elements of experience (*item‐specific information)* whereas the hippocampus establishes *relations* between them to bring about unique event representations [e.g., Mayes, Montaldi, & Migo, 2007]. Regions in the prefrontal cortex (PFC) modulate these encoding processes as a function of stimulus properties and task demands and during retrieval they orchestrate retrieval strategies and monitor their success. Contextually rich *recollection* is thought to ensue when the hippocampus (under the influence of PFC) successfully re‐establishes the spatial‐temporal relations that uniquely define a specific prior event, whilst a sense of *familiarity* prevails when information is retrieved through ErC and PrC processes that do not yield sufficient relational context to support the reconstruction of unique episodes [see Brown & Aggleton, 2001; Eichenbaum, 2004; Eichenbaum, Yonelinas, & Ranganath, 2007; Fletcher & Henson, 2001; Henson, 2005; Simons & Pierce, 2003; Spaniol, Davidson, Kim, Han, Moscovitch, & Grady, 2009, Squire, Wixted, & Clark, 2007 for reviews].

To date, relatively few studies have examined the neural underpinnings of memory decrements in ASD through imaging methods, with those that have focusing primarily on the domain of working memory. The evidence in this context suggests reduced involvement of prefrontal regions in the online maintenance of information over short (a few seconds) periods of time [Luna, et al., 2002; Koshino, Carpenter, Minshew, Cherkassky, Keller, & Just, 2005; see Brandse, et al., 2013 for a review]. Such abnormalities may contribute to difficulties over longer delays by hampering the generation of relations between elements of an episode, thus attenuating the tendency for contextually rich recollection at retrieval [Bigham, Boucher, Mayes, & Anns, 2010; Boucher, 2007; Bowler et al.^2014^, ^2007^]. A recent EEG experiment lends some support to this possibility by demonstrating that event related potentials (ERPs) associated with recollection are relatively undifferentiated from those associated with familiarity based retrieval in ASD [Massand, Bowler, Mottron, Hosein, & Jemel, 2013]. It remains unclear, however, to what extent these anomalies might reflect differences already at the stage of encoding. The present study examines this issue, by drawing on a paradigm by Addis and McAndrews [2006] who asked participants to study word‐triplets during fMRI scanning that varied in the number of conceptual relations between component words for a later recognition task. Their results suggested that the inferior frontal gyrus (IFG) of the PFC is involved in *generating* relational information when it is not obviously given by the stimulus, whilst the hippocampus *binds* available relations in the service of later retrieval [see also Lepage, Habib, Cormier, Houle, & McIntosh, 2000].

If difficulties in contextually rich recollection in ASD are, at least in part, mediated by difficulties in the encoding of relational information we would expect the following pattern of results on a task such as that by Addis & McAndrews [2006]. First, based on the view that relational encoding fosters subsequent contextually rich recollection we would expect that experiences of recollection but not familiarity would increase as a function of the number of conceptual relations in the to‐be‐remembered word triplets. Second, individuals with ASD would be expected to report fewer experiences of recollecting studied word triplets despite overall preserved levels of recognition memory. Third, the IFG encoding processes that have been linked to the generation of relational information should be attenuated in ASD. And fourth, the MTL processes typically associated with the binding of available relational information should also be attenuated in ASD.

## Materials and Methods

### Participants

Fourteen individuals with a diagnosis of ASD and fourteen typically developing (TD) comparison adults served as participants. Three individuals (1 ASD, 2 TD) were excluded from subsequent analyses because of inattention during encoding, failure to follow task instructions, or identification of neuropathology on a routine inspection of structural scans. All remaining individuals were free of medication and reported no family history of psychiatric or neurological disorders other than ASD. The experimental procedures were prospectively reviewed and approved by the National Research Ethics Service (Essex 2 Research Ethics Committee).

The final ASD group comprised 12 males and 1 female (all right handed) who were all diagnosed by local health professionals according to the 4th edition of the diagnostic and statistical manual of mental disorders (DSM‐IV) criteria (American Psychiatric Association, 2000]. Assessment with the Autism Diagnostic Observation Schedule [ADOS; Lord, et al., 1989) further supported these diagnoses. TD participants (11 males, 1 female; 1 left handed male) were matched to ASD participants on the basis of chronological age and Wechsler IQ [WAIS‐III^UK^; The Psychological Corporation, 2000] and were screened for characteristics that may be commensurate with a diagnosis of ASD using the Autism Spectrum Quotient questionnaire [ASQ; Baron‐Cohen, Wheelwright, Skinner, Martin, & Clubley, 2001]. Descriptive statistics for the two groups are summarized in Table 1.

**Table 1:** Descriptive Statistics for Participant Groups

Measure	ASD (*n* = 13)			TD (*n* = 12)			Cohen's *d*
	*M*	SD	Range	*M*	SD	Range	
Age (years)	35.6	10.3	22.6–55.5	35.5	10.5	22.9–54.5	0.01
VIQ	106.4	12.4	81–128	113.1	15.2	86–134	0.48
PIQ	107.3	17.6	84–136	108.0	13.8	81–125	0.04
FIQ	106.2	16.3	81–127	110.2	14.8	83–127	0.26
ASQ	34.5^a^	7.1	22–45	15.8^a^	4.9	8–22	3.07^a^
ADOS Com.	3.2	1.3	1–5	—	—	—	—
ADOS RSI.	7.2	2.5	3–12	—	—	—	—
ADOS Total	10.3	3.2	5–17	—	—	—	—

a (*t* = 7.07, df = 23, *P* < 0.001).

ASD and TD groups were well matched in terms of Age (in years), Verbal (VIQ), Performance (PIQ) and Full‐scale (FIQ) Wechsler intelligence quotients. The ASD group scored significantly (*t* = 7.07, df = 23, *P* < 0.001) higher on the Autism Spectrum Questionnaire (ASQ). Autism Spectrum Diagnostic Observation Schedule (ADOS) Communication (Com.), Reciprocal Social Interaction (RSI) and Total algorithm scores supported the diagnosis for ASD participants.

### Materials

**Figure 1:**
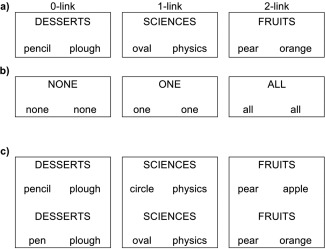
Examples of a) to‐be‐remembered encoding triads, b) control triads, and c) two‐alternative forced‐choice recognition items.

With the exception of minor amendments during test, the materials and procedures of this experiment were identical to those used by Addis and McAndrews [2006].^1^ Briefly, 108 word triads were constructed, each comprising a capitalized category label and two words in lower‐case font. Either none, one or both of these words were legitimate examples of the named category (36 triads each); hereafter, “0‐link,” “1‐link,” and “2‐link” triads, respectively. During the encoding scan 36 control triads, comprising the words “None,” “One,” or “All,” were randomly interspersed with target triads. For the two‐alternative forced choice recognition test that participants performed outside the scanner, encoded word triads were presented alongside lure triads on the top and bottom half of a laptop monitor. Lure triads differed from encoded triads only with respect to one of the lower‐case exemplar words, which was substituted with a conceptually related item. The position of target and lure triads on the screen and the left right position of substituted items in lure triads was counterbalanced across items. For half of the “1‐link” triads the lure triads substituted the legitimate category exemplar while for the remaining half the unrelated exemplar word was substituted. Figure 1 provides examples of the experimental materials.

### Procedure

Participants were familiarized with the encoding task outside the scanner through a series of 18 practice trials (12 encoding triads and 6 control triads). They were asked to indicate, via a keyboard response, whether “none,” “one,” or “all” of the exemplar words were legitimate examples of the named category (or to press the key corresponding to the words shown on the screen in the case of control triads). The timing of practice trials was identical to that to be used in the scanner with 6 sec per triad followed by a central fixation cross varying in duration between 4 and 8 sec to provide jitter. Response keys on the keyboard (“J,” “K,” and “L”) during practice were chosen to mimic the relative positions of the buttons on the response box in the scanner (index finger = “none”; middle finger = “one”; ring finger = “all”). Instructions clarified that memory for the triads would be tested afterwards although the nature of the memory test was not disclosed. Once participants indicated that they understood what was required, their written informed consent was obtained and they were prepared for scanning.

The scanning session lasted approximately 1 hr and began with a series of structural scans for an unrelated project. Experimental trials were presented in three 9.6 min functional runs comprising 36 encoding (12 of each link type) and 12 control triads each. The order of trials was random with respect to triad type but fixed across subjects. Stimuli were presented in black Arial font on a light‐grey background and back‐projected onto a white screen that participants viewed through a mirror mounted on the head‐coil. Responses were made through an MR‐compatible four‐button response box. Stimulus presentation and recording of participants' responses were controlled by in‐house software. Immediately after the final run, participants were taken to a quiet room where they first completed a 7 min nonverbal distracter task (mental rotation). Participants were then told that they would see all of the word triads they had studied in the scanner once more alongside similar triads they had not seen. The order of test trials was randomized for each participant, whose principal task was to decide which of the two triads they had seen earlier. Unlike Addis and McAndrews [2006] we gave participants unlimited time to make their decisions during this test and we also asked participants to qualify their choices using the “Remember/Know/Guess” procedure [Gardiner, Ramponi, & Richardson‐Klavehn, 2002]. Thus, participants indicated whether they recollected the study episode (“*Remember*”) for a particular triad, whether they were simply familiar with one of the triads (“*Know*”) or whether they were purely guessing (“*Guess*”*)*.

### fMRI Acquisition, Processing, and Analysis

Data were acquired on a 3.0T GE Signa system (General Electric Medical Systems) at the Institute of Psychiatry, King's College London. fMRI data were acquired through T2* weighted Gradient Echo sequences (TE = 30 ms, TR = 2000 ms, FOV = 240 mm) during which 38 slices (3 mm thick, 0.3mm gap), horizontally aligned to the AC‐PC line and covering the entire brain, were collected. All preprocessing and analyses were performed in SPM5 (Wellcome Department of Cognitive Neurology, UK) unless otherwise specified. Functional images were realigned for motion correction, slice‐time corrected, spatially normalized to an MNI template, and smoothed using a Gaussian kernel of 8 mm full‐width half maximum. Recognition performance during test was used to retrospectively classify each stimulus event during scanning as either a subsequently “Remembered,” “Known,” “Guessed,” or “Forgotten” (i.e., a triplet for which the participant chose the incorrect option during the forced‐choice test) word triad. These events, together with control triads and the participant's key‐presses, were modeled as fixed effects at the individual level using the canonical hemodynamic response function in SPM5 (head‐movement parameters were also included as regressors). Statistical parametric maps of the t‐statistic (SPM{t}) were generated for each subject and the contrast images were stored for further random‐effects analyses at the second level (see results for details). Only trials were modeled for which participants gave a correct response during the encoding task, to ensure that temporary lapses of concentration did not contaminate the analyses. To identify regions sensitive to the number of relational links in encoded triads, additional models were estimated that included linear parametric predictors [0 1 2]. Within and between‐group effects of interest were examined at the second level within full‐factorial random‐effects models.

Similar to Addis and McAndrews [2006] we focused our analyses primarily on anatomical regions of interest within bilateral IFG and MTL after confirming, at the whole brain level using stringent thresholds (*P* ≤ 0.005, whole‐brain FDR corrected, minimum extent threshold of 10 voxels) that these regions were indeed involved in the successful encoding of stimuli. To test for the specific within and between group effects of interest we used an uncorrected threshold of *P* ≤ 0.005 with a minimum extent threshold of 10 contiguously activated voxels. For these analyses a single ROI mask was generated using MARINA (Bender Institute of Neuroimaging; University of Giessen, Germany), comprising the hippocampi and parahippocampal gyri of the MTL and the opercular as well as triangular parts of the IFG bilaterally. Anatomical locations of observed signal contrasts are reported using the Talairach coordinate system and anatomical labels were obtained with the aid of the Talairach client [Lancaster, et al., 2000]. Percent signal changes were extracted and averaged from all the suprathreshold voxels of first‐level contrasts that fell within the region of suprathreshold voxels at the second level using the rfxplot toolbox for SPM5 [Gläscher, 2009]. Similar to the MarsBaR toolbox [Brett, Anton, Valabregue, & Poline, 2002], the rfxplot toolbox computes percent signal changes relative to the voxel‐wise baseline (i.e., the mean signal within the selected voxels) rather than a whole‐brain baseline.

## Results

### Behavioral Data

Table 2 summarizes the reaction time and accuracy data for participants' responses during the encoding runs. Reaction time data for 1 individual in the TD group were not available due to a misunderstanding of the instructions (a response was given after rather than during triad presentation). A 2 (Group: ASD vs. TD) × 3 (Triad Type: 0‐link vs. 1‐link vs. 2‐link) analysis of variance (ANOVA) of reaction times yielded a main effect of Triad Type (*F*(2,44) = 9.55, *P* < 0.001) that was due to slower responses during 0‐link than 1‐link (*t =* 2.79, df *=* 24, *P* < 0.05) or 2‐link triads (*t =* 3.84, df *=* 24, *P* < 0.01), which in turn did not differ significantly from one another (*t =* 1.54, df *=* 24, *P* = 0.14). Although there was no overall group effect (*F*(1,22) =.15, *P* = 0.69), there was a Group × Triad Type interaction (*F*(2,44) = 3.31, *P* < 0.05), whereby TD participants responded fastest during 1‐link triads while ASD participants responded fastest during 2‐link triads. Response accuracy was also characterized by a main effect of Triad Type (*F*(2,44) = 15.24, *P* < 0.001) and a Group × Triad Type interaction (*F*(2,44) = 5.71, *P* < 0.01) in the absence of a main effect of Group (*F*(1,22) = 2.17, *P* = 0.16). This pattern was the result of participants generally responding most accurately to 0‐link triads and least accurately to 2‐link triads with the ASD group performing worse than the TD group on 2‐link triads (*t =* 2.89, df *=* 22, *P* < 0.01) but not 0‐link (*t =* 1.00, df *=* 22, *P* = 0.33) or 1‐link triads (*t =* .28, df *=* 22, *P* = 0.78). Considered together, these results do not suggest gross differences in encoding performance between groups.

**Table 2:** Reaction Time and Accuracy During the Encoding Task in the Scanner

	ASD		TD		Cohen's *d*
	*M*	SD	*M*	SD	
Reaction Time (ms)					
0‐link	2880	532	2795	508	0.16
1‐link	2789	599	2563	443	0.43
2‐link	2567	618	2628	501	0.13
Accuracy					
0‐link	0.95	0.06	0.97	0.03	0.42
1‐link	0.93	0.08	0.94	0.07	0.13
2‐link	0.87	0.06	0.94	0.05	1.27

Both groups performed the encoding task (i.e., deciding how many exemplar words were valid members of the named category) at near ceiling levels of accuracy with ASD participants committing somewhat more errors for 2‐link triads.

**Figure 2:**
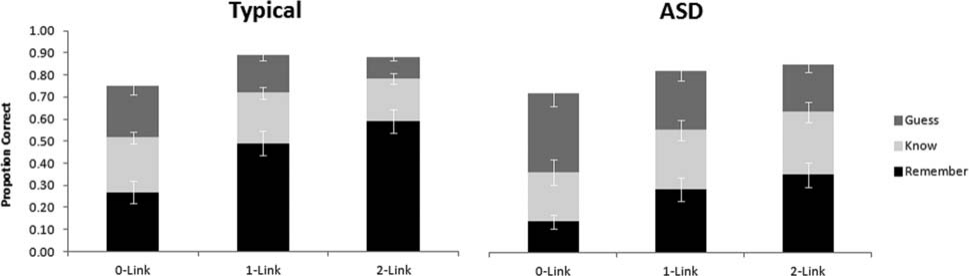
Average proportion of “Remember” (black), “Know” (light grey), and “Guess” (dark grey) responses that make up the correct choices during the 2 alternative‐forced‐choice recognition test for ASD and TD groups as a function of triad type (Error bars represent 1 standard error). Despite overall equivalent correct recognition performance in the two groups, the data replicate previous observations of selectively attenuated Remembering in the ASD group. It is also evident that only Remember responses increase as a function of the number of relational links.

Performance on the forced‐choice recognition test following the scan is set out in Figure 2. A 2 (Group: ASD vs. TD) × 3 (Triad Type: 0‐link vs. 1‐link vs. 2‐link) × 3 (Recognition Judgment: Remember vs. Know vs. Guess) mixed ANOVA of these data revealed main effects for Trial Type (*F*(2,46) = 26.40, *P <* 0.001) and Recognition Judgment (*F*(2,46) = 5.06, *P <* 0.05), with better performance on 1‐link (*t =* 5.69, df *=* 25, *P* < 0.001) and 2‐link (*t =* 6.30, df *=* 25, *P* < 0.001) as compared to 0‐link trials and overall more Remember than Know (*t* = 4.44, df = 25, *P* < 0.001) and more Know than Guess responses (*t* = 8.62, df = 25, *P* < 0.001). More importantly, we observed the predicted interaction between Group and Recognition Judgment (*F*(2,46) = 6.10, *P <* 0.01), which replicates earlier demonstrations of attenuated “Remembering” in ASD (*t* = 2.95, df = 23, *P* < 0.01) despite overall preserved levels of recognition memory [e.g. Bowler, et al., 2007]. In addition, the data were characterized by the expected Recognition Judgment × Triad Type (*F*(4,92) = 26.40, *P <* 0.001) interaction whereby “Remember” responses increased as a function of the number of category relations in word triads (*F*(2,50) = 49.68, *P <* 0.001) while “Know” responses were unaffected (*F*(2,50) = 0.10, *P =* 0.90) and “Guess” responses decreased (*F*(2,50) = 21.04, *P <* 0.001). This interaction confirms that recollection, as indexed by “Remembering” at retrieval, is strongly associated with the processing of relational information during encoding.

### fMRI Results

#### Successful encoding effects

To identify regions involved in the successful encoding of stimuli in ASD and TD participants, the fixed‐effects models generated for each participant at the first level of the SPM analysis were contrasted as random‐effects at the second level using a 2 (ASD vs. TD) × 2 (Combined Remember and Know vs. Baseline Triads) whole‐brain ANOVA (*P* ≤ 0.005, whole‐brain FDR corrected, minimum extent threshold of 10 voxels). In line with Addis and McAndrews [2006] there was evidence of robust bilateral activation of PFC and MTL regions during successfully encoded (Remembered and Known) as compared to baseline triads. This observation held for both groups individually and a conjunction analysis showed considerable group overlap, including in clusters of the left IFG and the middle segment of the left hippocampus (see Table 3 for details). Somewhat unexpectedly, a group comparison of this contrast within our anatomical ROIs revealed a more pronounced successful encoding contrast in ASD as compared to TD individuals in left IFG (BA45; *x* = −51, *y* = 24, *z* = 8; *z*‐score = 2.90; *P* < 0.005). No group differences were apparent in the MTL, unless the statistical criterion was relaxed to *P* < 0.01 (maintaining a minimum extent threshold of 10 contiguous voxels). At this threshold, and in line with predictions, the successful encoding signal in a posterior region of the left hippocampus (*x* = −31, *y* = −38, *z* = −5; *z*‐score = 2.57) was enhanced in TD as compared to ASD participants.

**Table 3:** Brain Regions Associated with Successful Encoding Processes in Both ASD and TD Groups

Brain Regions	Talairarch			*z*‐score
	*x*	*y*	*z*	
L Lingual Gyrus (BA 18)	−18	−94	−14	6.24
L Middle/IFG (BA 46/47)	−44	15	23	5.96
L Cerebellum	−42	−61	−24	5.79
L Parahippocampal/Hippocampus	−30	−17	−14	4.53
L Precentral Gyrus (BA 4)	−42	−10	53	4.27
L Fusiform Gyrus (BA 37)	−47	−40	−8	3.93
R Inferior Occipital Gyrus (BA 17)	21	−93	−6	6.67
R Cerebellum	34	−67	−23	4.34
R Insula (BA13)	31	25	0	3.68

A conjunction analysis of ASD and TD groups identified the tabulated regions as significantly involved in successful encoding processes (i.e., combined Remember & Know versus Baseline triad contrast) in both participant groups (*P* < 0.005, whole‐brain FDR corrected; minimum extent 10 contiguous voxels).

#### Remember/know effects

**Figure 3:**
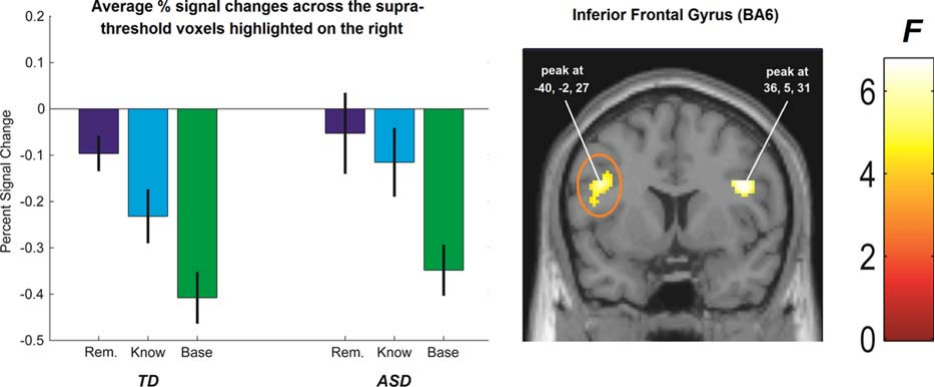
Voxels in left and right inferior prefrontal cortex that are sensitive to a Group × Recognition Judgement interaction (*P* < 0.005; uncorrected with a minimum extent threshold of 10 contiguous voxels). Average percent signal changes (relative to the voxel‐wise baseline) are shown across all voxels of the left IFG region that are sensitive to this interaction as a function of group (Left set of bars = TD; Right set of bars = ASD) and subsequent recognition judgement (Remembered vs. Known; baseline trials are shown for comparison)—Error Bars represent 1 standard error. A successful encoding effect (i.e., combined Remember & Know > Baseline) is evident in both groups but significant differences between subsequently recollected versus familiar word triads are aparent only in the TD but not the ASD group.

To examine the above group differences in encoding processes more closely, and to begin to establish how they might underpin diminished recollection in ASD at retrieval, we first modeled the main effect of recognition judgment (Remember > Know) across both groups, followed by the interaction between recognition judgment and group in a 2 (Group) × 2 (Remember vs. Know) full‐factorial ANOVA. In line with previous observations [e.g., Ranganath, Yonelinas, Cohen, Dy, Tom, & D'Esposito, 2003; see Kim, 2011 for a review] robust clusters extending over large areas of the middle and inferior frontal gyri exhibited increased signal changes during the encoding of items subsequently “Remembered” as opposed to “Known” (BA46; *x* = −44, *y* = 16, *z* = 19; *z*‐score = 3.34 and *x* = −40, *y* = 30, *z* =10; *z*‐score = 2.87; *P* < 0.005). When examining the groups separately this pattern was reliable only for TD participants and when the Group × recognition judgment interaction was modeled directly, two clusters in the left (BA6; *x* = −40, *y* = −2, *z* = 27; *z*‐score = 3.67; *P* < 0.005) and right (BA6; *x* = 36, *y* = 5, *z* = 31; *z*‐score = 3.61; *P* < 0.005) IFG were identified. As Figure 3 illustrates, this interaction is the result of robust signal differentiation between subsequently Remembered versus Known word triads in TD but not ASD participants, which, incidentally, helps to explain why the successful encoding contrast in the ASD group in the analysis above was enhanced overall. In other words, because groups did not differ in relation to signal changes related to baseline triads (shown for comparison in Fig. 3), the reduced signal differentiation between “Remembered” versus “Known” triads in ASD participants essentially augments the overall successful encoding contrast in comparison to TD participants. Turning to the MTL regions of interest, no reliable Remember versus Know signal contrasts or group differences in such contrasts were observed and no regions in either IFG or MTL demonstrated enhanced signal changes for subsequently “Known” over “Remembered” word triads.

#### Parametric effects of the number of categorical links in word triads

**Figure 4:**
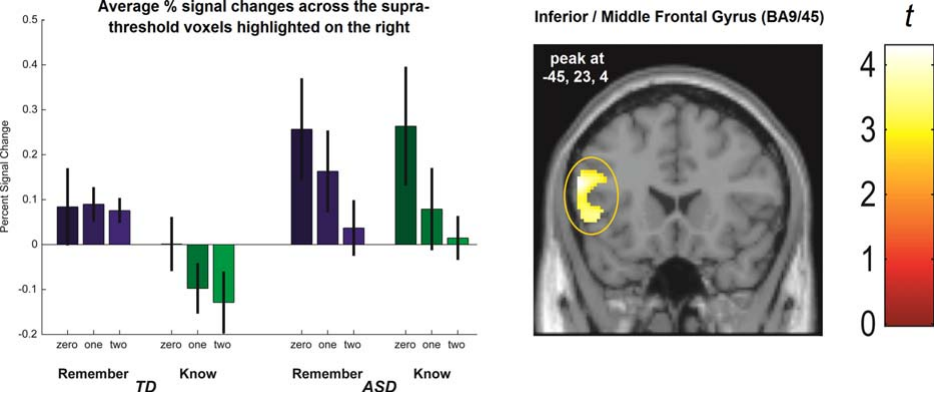
Parametric modulation of percent signal changes (relative to voxel‐wise baseline) in Inferior prefrontal cortex as a function of participant group (Left set of bars = TD; Right set of bars = ASD), recognition judgement (Remembered triads purple; Known triads green) and the number of relational links in word triads (zero link, one link, and two link triads). The coronal section illustrates the voxel cluster that is sensitive to the inverse association between prefrontal signal changes and relational links in word triads across both groups of participants (*P* < 0.005; uncorrected with a minimum extent threshold of 10 contiguous voxels). As the barchart shows, however, this inverse relationship in TD participants was only observed for subsequently known but not remembered word triads, whereas in the ASD group the liniar decrease was robust irrespective of subsequent recognition judgement. Error bars represent 1 standard error.

The evidence above suggests that the encoding of subsequently “Remembered” versus “Known” word triads is subserved by distinguishable neural processes in TD but not ASD participants in regions of the prefrontal cortex. To shed further light on this pattern, we next examined the parametric modulation of encoding related signal changes in this area as a function of the number of relational links in word triads. For this analysis, we subjected the linear parametric predictors (with three levels identifying 0‐link, 1‐link, and 2‐link triads) entered at the first level to a 2 (Group) × 2 (predictor associated with Remembered vs. Known triads) full‐factorial ANOVA at the second level. In line with Addis and McAndrews [2006], this analysis confirmed, irrespective of recognition judgment and across both groups, a negative association between relational links and signal changes in the left IFG (BA 45/9; *x* = −45, *y* = 23, *z* = 4; z‐score = 3.90; *P* < 0.005). When examining this association for the two groups separately, it was found to be reliable only for ASD but not TD participants. The source of this somewhat surprising observation became apparent when we extracted % signal changes, which are set out in Figure 4 as a function of group, recognition judgment (Remember vs. Know) and relational links (zero, one, or two). As the data illustrate, in the ASD group signal changes decreased as a function of relational links irrespective of whether triads were subsequently “Remembered” or “Known.” In the TD group by contrast, the negative association between signal changes and relational links was apparent only for subsequently “Known” but not “Remembered” triads. Unlike Addis & McAndrews [2006], we did not observe the predicted positive association between signal changes in the hippocampus and the number of relational links in word triads.

## Discussion

Based on existing behavioral evidence concerning memory functioning in ASD it remains unclear whether difficulties in this domain stem from atypicalities at the stage of encoding, retrieval or both. We tested the prediction that difficulties in recollection in ASD are, at least in part, attributable to anomalies in the encoding of relational information, and the behavioral data provided clear support for this prediction. Specifically, recollection but not familiarity based recognition was compromised in ASD [e.g., Bowler et al^2014^, ^2007^] and only recollection but not familiarity increased as a function of the number of relational links in the studied word‐triads. Together, these observations confirm that recollection at retrieval is closely linked to the processing of relational information at encoding and they lend support to the suggestion that attenuated recollection in ASD is in part attributed to anomalies in relational encoding processes.

At the neural level, the present results offer an independent replication of Addis & McAndrews [2006] observation that signal changes in the inferior frontal region of the left PFC generally increase as a function of decreasing category relations in to‐be‐remembered word‐triads, supporting the notion that this region is important for the generation of relational information when this is not immediately given by the stimulus environment. The observations also confirm previous demonstrations of robust signal differentiation in the IFG for subsequently recollected versus familiar stimuli [e.g., Ranganath, et al., 2003]. Although hippocampal activity was related to later retrieval success, the results did not replicate Addis & McAndrews [2006] observations of a positive association between hippocampal signal changes and the number of category relations in to‐be‐remembered triplets that would underscore the role of the hippocampus in relational binding processes [Mayes et al., 2007]. Since the sample size, and scanning parameters are very comparable between the current and Addis & McAndrews [2006] original study, the most likely source for these discrepancies are the changes we implemented to the recognition procedure. Specifically, participants had unlimited time to respond during the recognition test (compared to the original 6s time limit) and they were required to qualify their choices as either “Remembered,” “Known,” or “Guessed.” This may have led participants to engage more elaborate and varied recognition strategies that potentially obscured some of the hippocampal effects that would otherwise be driven by stimulus characteristics.

In ASD, we expected the prefrontal and medial‐temporal processes mediating the generation and binding of relational information to be attenuated. In line with predictions, the data indicated somewhat reduced engagement of a left posterior hippocampal region in the ASD group, which suggests anomalies in relational binding processes. This observation, however, merits replication in larger samples. In relation to prefrontal processes the prediction of attenuated or atypically modulated encoding processes in ASD was clearly not confirmed. Instead the successful encoding contrasts were overall enhanced in the PFC in the ASD group, and signal changes in this region demonstrated a robust inverse relation with the number of category relations in to‐be‐remembered word triads. Increases in prefrontal activity during memory formation have also been observed in the elderly [Miller et al., 2008; Presson et al., 2006], where they are thought to reflect the engagement of more effortful encoding processes that compensate for age‐related structural and/or functional declines in memory networks. Structural and functional PFC abnormalities are widely reported in the ASD literature [e.g., Duerden, Mak‐Fan, Taylor, & Roberts, 2012] and behaviorally some parallels have been noted between the memory profile seen in ASD and that seen in older adults [see Bowler & Gaigg, 2008] and patients with frontal lobe pathology [e.g., Bowler et al.^2010^, ^2014^; Steele, et al.^2014^, ^2007^]. Thus, it seems highly likely, that the enhanced successful encoding contrast observed in the current study, is a reflection of the engagement of more effortful encoding strategies, possibly to compensate for attenuated hippocampal binding processes.

Besides the overall enhanced encoding related PFC activation in ASD, there was also a relative lack of signal differentiation between subsequently recollected versus familiar word triads in this group. This may simply be a corollary of more effortful encoding processes in ASD, which could result in a ceiling‐type effect within the PFC whereby each triplet is processed with the maximum resources available. This seems unlikely, however, since PFC activation is clearly sensitive to the number of conceptual relations available for processing, and ceiling effects should attenuate also these effects. Another possibility is that ASD constitutes an example of a single dissociation of functions where one of two processes is either absent or so significantly compromised that the other process dominates behavior. This suggestion was first put forward by Massand et al., [2013] who observed that in TD participants, temporally and topographically distinct ERP components are associated with item (line drawings) versus associative (line drawing—color association) recognition judgments during retrieval whereas in an ASD group the same ERP components were associated with both types of recognition judgments. In previous behavioral studies, we have shown that memory encoding processes in ASD tend to be biased to the processing of item‐specific information, whereas TD participants tend to process relational as well as item‐specific information in parallel [Gaigg et al., 2008; Bowler et al.^2009^, ^2014^]. Thus, the neural observations in the current study and in Massand et al. [2013] may be a reflection of a processing bias for item‐specific information in ASD. Because such a bias would be less optimal, it is likely to require greater effort. In other words, a processing bias could account for both the overall greater PFC engagement during encoding as well as the relative lack of signal differentiation as a function of subsequent recognition judgment.

Importantly, and in line with the “Task Support Hypothesis” [Bowler et al.^2014^, ^2004^], previous behavioral work suggests that certain encoding conditions that promote the explicit processing of relational information can ameliorate memory difficulties in ASD [Gaigg et al., 2008; Bowler et al., 2010] similar to how retrieval support does. It would be of interest for future studies to establish in how far such encoding conditions also “normalise” neural encoding processes, since any conditions that do may be utilized fruitfully by educators and practitioners to ameliorate behavioral difficulties in the domain of learning and memory in ASD and also promote the development of neural circuitry that may not mature typically without targeted support.

## Footnotes

1 We thank the authors for kindly providing copies of their materials.
